# Dopamine D_4_ Receptor Counteracts Morphine-Induced Changes in μ Opioid Receptor Signaling in the Striosomes of the Rat Caudate Putamen

**DOI:** 10.3390/ijms15011481

**Published:** 2014-01-21

**Authors:** Diana Suárez-Boomgaard, Belén Gago, Alejandra Valderrama-Carvajal, Ruth Roales-Buján, Kathleen Van Craenenbroeck, Jolien Duchou, Dasiel O. Borroto-Escuela, José Medina-Luque, Adelaida de la Calle, Kjell Fuxe, Alicia Rivera

**Affiliations:** 1 Department of Cell Biology, School of Science, University of Málaga, 29071 Málaga, Spain; E-Mails: boomgaard@uma.es (D.S.-B.); ale_valde@uma.es (A.V.-C.); rrb@uma.es (R.R.-B.); medi_luque@hotmail.com (J.M.-L.); delacalle@uma.es (A.C.); 2 Department of Neuroscience, Biodonostia Institute, 20014 San Sebastián, Spain; E-Mail: belen.gago@biodonostia.org; 3 Laboratory of Eukaryotic Gene Expression and Signal Transduction (LEGEST), Ghent University-Gent, 9000 Ghent, Belgium; E-Mails: kathleen.vancraenenbroeck@ugent.be (K.V.C.); Jolien.duchou@ugent.be (J.D.); 4 Department of Neuroscience, Karolinska Institutet, Retzius väg 8, 17177 Stockholm, Sweden; E-Mails: Dasiel.Borroto-Escuela@ki.se (D.O.B.-E.); Kjell.Fuxe@ki.se (K.F.)

**Keywords:** morphine, PD168,077, μ opioid receptor, dopamine D_4_ receptor, G proteins, caudate putamen, striosomes, addiction

## Abstract

The mu opioid receptor (MOR) is critical in mediating morphine analgesia. However, prolonged exposure to morphine induces adaptive changes in this receptor leading to the development of tolerance and addiction. In the present work we have studied whether the continuous administration of morphine induces changes in MOR protein levels, its pharmacological profile, and MOR-mediated G-protein activation in the striosomal compartment of the rat CPu, by using immunohistochemistry and receptor and DAMGO-stimulated [^35^S]GTPγS autoradiography. MOR immunoreactivity, agonist binding density and its coupling to G proteins are up-regulated in the striosomes by continuous morphine treatment in the absence of changes in enkephalin and dynorphin mRNA levels. In addition, co-treatment of morphine with the dopamine D_4_ receptor (D_4_R) agonist PD168,077 fully counteracts these adaptive changes in MOR, in spite of the fact that continuous PD168,077 treatment increases the [^3^H]DAMGO *B*_max_ values to the same degree as seen after continuous morphine treatment. Thus, in spite of the fact that both receptors can be coupled to G_i/0_ protein, the present results give support for the existence of antagonistic functional D_4_R-MOR receptor-receptor interactions in the adaptive changes occurring in MOR of striosomes on continuous administration of morphine.

## 1. Introduction

The opioid morphine is one of the most potent analgesic drugs used to relieve moderate to severe pain [1]. After long-term use of morphine, neuroadaptive changes in the brain promotes tolerance, which result in a reduced sensitivity to most of its effects with attenuation of analgesic efficacy, and dependence, revealed by drug craving and physical or psychological manifestations of drug withdrawal [2]. Alongside tolerance and dependence, behavioral sensitization develops after repeated intermittent treatment with morphine, which is characterized by an increase of responsiveness to the same or lower doses of the drug [3]. Morphine research has long been focused on the development of analogs, or drug administration strategies, which could result in an effective analgesic therapy without side effects.

Opioids exert their pharmacological actions through their interactions with the opioid receptors μ (MOR), δ (DOR), and κ (KOR), which belong to the family of G protein-coupled receptors (GPCRs) [4]. The studies using MOR knockout mice have revealed that this receptor is critical, not only in mediating morphine analgesia, but also in addictive behaviors by the induction of a strong rewarding effect [5–7]. On the other hand, KOR function opposes to the action of MOR and it is considered as the major anti-reward system [8,9], whereas DOR contributes to contextual learning rather than opioid reward [10]. However, nowadays it is known that the regulation of these functions is more complex than expected due to the formation of GPCR heteromers. The existence of MOR-DOR and MOR-KOR heteromers has been demonstrated in the central nervous system (CNS) to integrate antinociceptive signals, having also a role in the addictive effect of opioids, such as morphine [11–13]. In addition, the endogenous opioids enkephalin (Enk) and dynorphin (Dyn), as the main ligands of these receptors in the CPu, can contribute to receptor regulation and downstream signaling processes [14].

The rewarding effects of morphine occur, in part, because MOR promotes dopamine release in the nucleus accumbens (NAc) and caudate putamen (CPu) [15,16]. The classical model of Johnson and North [17] postulates that morphine interacts with MOR located in GABAergic interneurons in the ventral tegmental area (VTA) and substantia nigra *pars* reticulata (SNr), leading to a disinhibition of mesencephalic dopamine neurons and an increase of the neural firing and dopamine release in the striatum. However, the cellular organization and regulation of these mesencephalic dopamine neurons is more complex than previously assumed [18], in addition, they also receive GABAergic inputs from terminals arising from extrinsic neurons. This is the case, for example, in the medium spiny GABA neurons (MSN) of the highly MOR enriched striosomal compartment of the CPu [19], which provide direct GABA inputs into the dopamine neurons of the substantia nigra *pars* compacta (SNc) [20,21].

Down-regulation of MOR or loss of MOR mediated G protein activity on effector responses have been proposed to occur during prolonged exposure to morphine leading to the development of tolerance [22]. However, contradictory results referred on MOR levels have been obtained in different regions of the CNS, including the CPu, from unchanged to increased expression [23–25], which has been related to morphine sensitization. We have previously demonstrated that the acute activation of the dopamine D_4_ receptor (D_4_R) decreases MOR immunoreactivity (IR) in the striosomal compartment of the CPu [26], where a high degree of co-localization between the two receptors exists [27]. Additionally, specific agonist activation of D_4_R prevents striatal acute and chronic morphine induced increases of several transcription factors (c-Fos, ΔFosB, and P-CREB) [28,29]. These data suggest the existence of antagonistic D_4_R-MOR interactions, probably occurring through the formation of receptor heteromers [11,30].

In the present work, we have studied the effect of D_4_R activation on MOR changes induced by morphine in the rat CPu on a continuous drug treatment paradigm, by analyzing MOR protein level, pharmacological profile, and functional coupling to G proteins. Furthermore, the levels of the endogenous opioids Enk and Dyn mRNA have also been determined.

## 2. Results

### 2.1. Cross-Inhibition of MOR IR Expression after Continuous Co-Administration of Morphine and the D_4_R Agonist PD168,077

**Figure 1.**
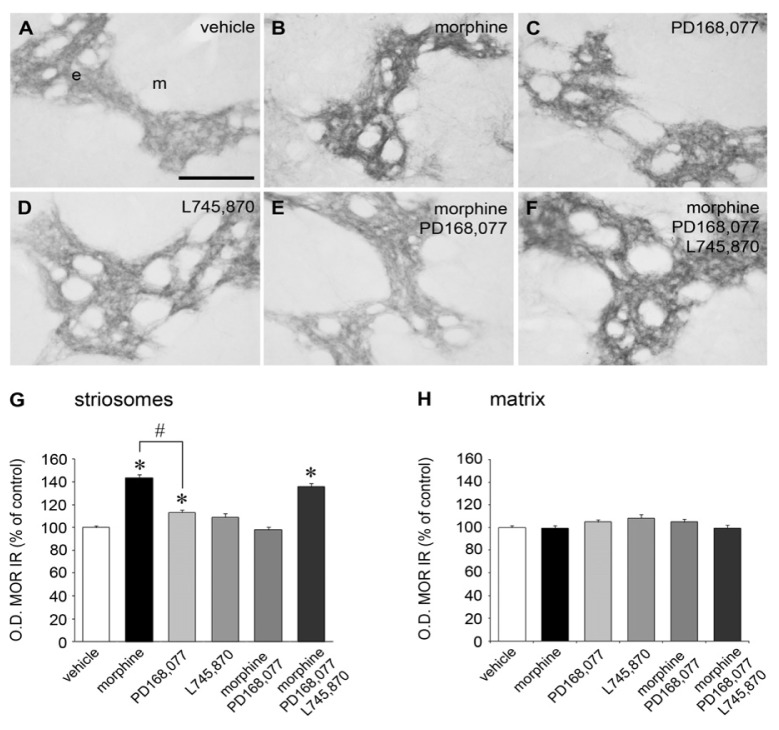
The D_4_R agonist PD168,077 counteracts MOR IR up-regulation in the striosomes of the rat CPu induced by the continuous treatment with morphine. (**A**–**F**) Representative photomicrographs showing MOR immunolabeled striosomes in the CPu from rats which received six days of continuous treatment with either vehicle (**A**); morphine (20 mg/kg/day) (**B**); PD168,077 (1 mg/kg/day) (**C**); L745,870 (1 mg/kg/day) (**D**); morphine + PD168,077 (**E**); or morphine + PD168,077 + L745,870 (**F**); Abbreviations: e, striosomes; m, matrix. Scale bar is 200 μm; (**G**,**H**) Effect of continuous drug treatments on MOR IR in the striosomes (**G**) and matrix (**H**) compartments, evaluated by determination of optical density (O.D.) values. Data represent mean ± SEM (*n* = 5) and are expressed as percentage of control. Differences between groups were set by one-way ANOVA followed by *post hoc* Bonferroni *t*-test. * *p* < 0.05 *vs.* control; # *p* < 0.05 morphine *vs.* PD168,077.

Levels of MOR protein expression were determined in the rat CPu after six days of continuous administration of morphine (20 mg/kg/day). Morphine significantly increased MOR immunoreactivity (IR) (by 44%) in the striosomes, but not in the matrix compartment (Figure 1A,B,G,H). Throughout the rostro-caudal axis, morphine produced a greater increase of MOR IR at caudal than at rostral and middle levels of the CPu (rostral: by 35%; middle: by 34%; caudal: by 69%) (Table 1).

A weak but significant increase in MOR IR (by 10%) was also observed in the striosomes of the CPu after continuous administration of the D_4_R agonist PD168,077 (1 mg/kg/day) (Figure 1A,C,G,H). This increase occurred exclusively in the striosomes of the caudal CPu (Table 1). When PD168,077 was administered at the same time with morphine, a complete suppression of morphine-induced rise of MOR IR was observed (Figure 1A,B,E,G,H). The blocking effect of the D_4_R agonist occurred at the rostral, middle, and caudal levels of the CPu (Table 1).

To test the specificity of the counteractive effect of PD168,077 on MOR IR, treatment with the D_4_R antagonist L745,870 was performed alone or in combination with morphine + PD168,077. Prolonged exposure to L745,870 (1 mg/kg/day) did not change MOR IR levels, neither in the striosomes nor in the matrix compartment (Figure 1A,D,G,H), independent of their location along the rostro-caudal axis of the rat CPu (Table 1). However, when the D_4_R antagonist L745,870 was co-administered with morphine + PD168,077, a 36% increase of MOR IR in the striosomes was observed, similar to that obtained in the morphine treated group (Figure 1A,B,F,G,H). Thus, the inhibitory action of PD168,077 was blocked. This action occurred especially in the caudal CPu, which showed the greatest increase in MOR IR (rostral: by 36%; middle: by 27%; caudal: 49%) (Table 1).

### 2.2. D_4_R Activation Counteracts the Increase of MOR Recognition and Signaling Induced by the Continuous Treatment with Morphine

**Figure 2.**
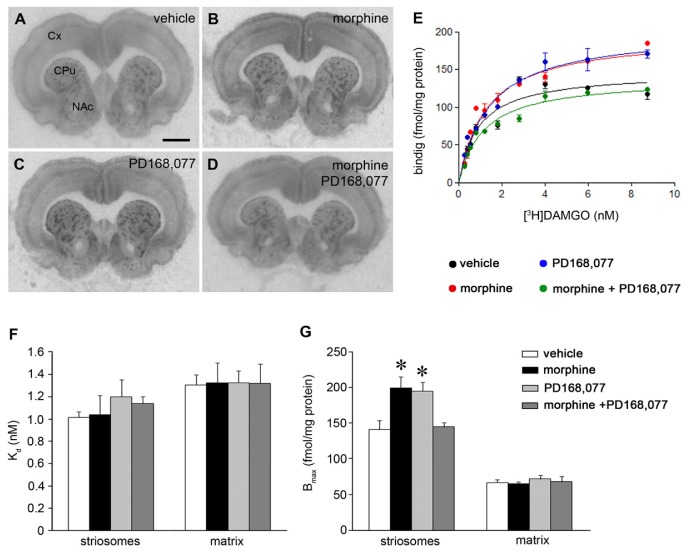
Co-administration of PD168,077 during continuous morphine treatment prevents the increase of [^3^H]DAMGO binding sites induced by the opioid drug. (**A**–**D**) Representative autoradiograms from coronal brain sections at the CPu level of rats which received six days of continuous treatment with vehicle (**A**), morphine (20 mg/kg/day) (**B**); PD168,077 (1 mg/kg/day) (**C**) and morphine + PD168,077 (**D**); Abbreviations: Cx, cortex; CPu, caudate putamen; NAc, nucleus accumbens. Scale bar is 2 mm. (**E**) Saturation curves of [^3^H]DAMGO binding in the striosomes; (**F**,**G**) Effect of drug treatments on *K*_d_ and *B*_max_ values (mean ± SEM; *n* = 6) of [^3^H]DAMGO binding in the striosomes and matrix compartment of the rat CPu. * *p* < 0.05 *vs.* control and morphine + PD168,077 (one-way ANOVA followed by *post hoc* Bonferroni *t*-test).

We, next, investigated the role of morphine and the D_4_R agonist PD168,077 in the regulation of MOR agonist binding in the CPu. First, the receptor binding characteristics of the MOR agonist [^3^H]DAMGO were compared in striatal sections from six-day-treated rats with morphine and/or PD168,077 (Figure 2A–E). The affinity of [^3^H]DAMGO (*K*_d_ value) was not affected by the different drug treatments (Figure 2F), neither in the striosomes nor in the matrix. However, both morphine and PD168,077 similarly increased the density of [^3^H]DAMGO agonist binding sites in the striosomes, which was reflected by an increase of *B*_max_ values (morphine, by 41%; PD168,077, by 38%) (Figure 2G). The co-administration of morphine and PD168,077 blocked these increases and resulted in a *B*_max_ value similar to that obtained in the vehicle treated group (Figure 2G).

**Figure 3.**
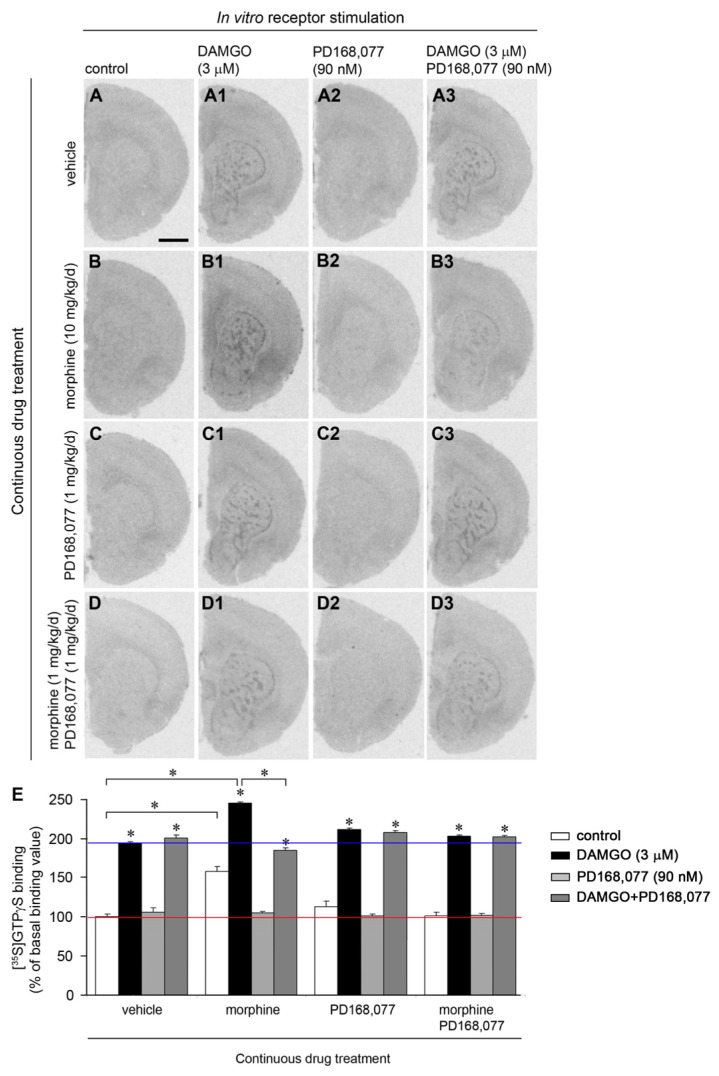
D_4_R *in vitro* activation prevents morphine-induced changes on MOR-dependent [^35^S]GTPγS binding. (**A**–**D3**) Representative autoradiograms of [^35^S]GTPγS binding in coronal sections of rat brain at the CPu level. Rats were continuously treated with vehicle (**A**–**A3**); morphine (20 mg/kg/day) (**B**–**B3**); PD168,077 (1 mg/kg/day) (**C**–**C3**) and morphine + PD168,077 (**D**–**D3**); Basal levels of [^35^S]GTPγS binding was determined in control sections from the four treatment groups (**A**,**B**,**C**,**D**) and *in vitro* receptor stimulation was performed with DAMGO (3 μM) (**A1**,**B1**,**C1**,**D1**); PD168,077 (90 nM) (**A2**,**B2**,**C2**,**D2**) or DAMGO + PD168,077 (**A3**,**B3**,**C3**,**D3**). Scale bar is 2 mm; (**E**) Effect of continuous drug treatments on [^35^S]GTPγS binding in the rat CPu after *in vitro* agonist stimulation of MOR and/or D_4_R. Data represent mean ± SEM (*n* = 6) and are expressed as percentage of basal [^35^S]GTPγS binding value in vehicle-treated animals (red line). Blue line represents DAMGO-dependent [^35^S]GTPγS binding in vehicle-treated animals. Differences between groups were set by two-way ANOVA followed by *post hoc* Bonferroni *t* test. * *p* < 0.05.

We also analyzed whether the activation of D_4_R alters the coupling of MOR to G proteins. In vehicle treated animals, *in vitro* application of the selective MOR agonist DAMGO (3 μM) resulted in an increase of [^35^S]GTPγS binding in the striosomes of the CPu (by 95%) (Figure 3A,A1,E), whereas no alteration was observed after *in vitro* incubation with PD168,077 (90 nM) (Figure 3A,A2,E). Co-stimulation of both MOR and D_4_R resulted in an increase of [^35^S]GTPγS binding in the striosomes (by 100%) similar to that observed in the DAMGO-stimulated control sections (Figure 3A,A1,A3,E).

Rats, which were continuously administered with morphine (20 mg/kg/day) for six days, showed a higher basal value of [^35^S]GTPγS binding (by 58%) in the whole CPu *vs.* vehicle treated rats (Figure 3A,B,E). MOR stimulation with DAMGO yielded an increase in [^35^S]GTPγS binding in the striosomes compared with DAMGO stimulation in vehicle-treated rat (Figure 3A1,B1,E). In these animals, *in vitro* co-application of DAMGO + PD168,077 restored [^35^S]GTPγS binding values to those observed in their paired control sections (Figure 3B,B3,E), but displaying a patchy distribution. Finally, animals which were continuously treated with PD168,077 (1 mg/kg/day) or morphine + PD168,077 showed a pattern of [^35^S]GTPγS binding after MOR and/or D_4_R *in vitro* stimulation similar to that described in vehicle treated animals (Figure 3C–C3,D–D3,E). Thus, the higher basal value found in rats treated with morphine alone was blocked by the combined morphine and D_4_R agonist treatment.

### 2.3. Absence of Changes in Enk and Dyn mRNA Levels in the CPu after the Continuous Administration of Morphine and/or PD168,077

**Figure 4.**
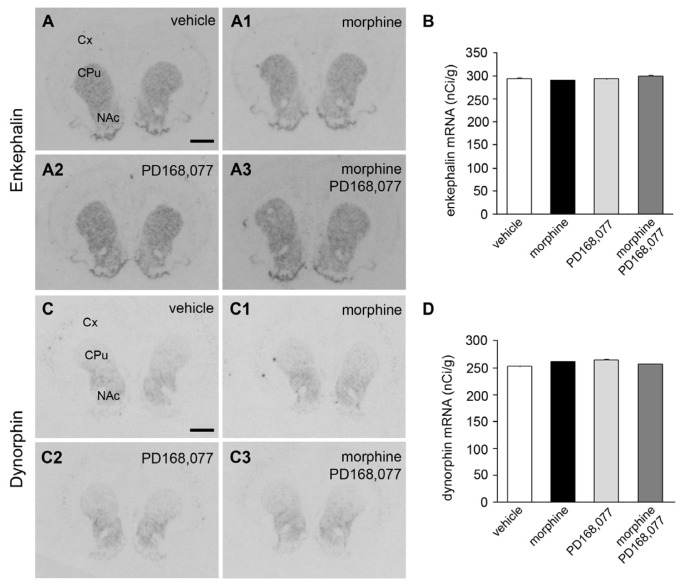
Lack of effect of continuous administration of morphine and/or PD168,077 on Enk and Dyn mRNA levels in the rat CPu. (**A**–**A3**,**C**–**C3**) Representative autoradiograms showing Enk (**A**–**A3**) and Dyn (**C**–**C3**) mRNA pattern of expression in the rat CPu after the administration of vehicle (**A**,**C**); morphine (20 mg/kg/day) (**A1**,**C1**); PD168,077 (1 mg/kg/day) (**A2**,**C2**) and morphine + PD168,077 (**A3**,**C3**); Abbreviations: Cx, cortex; CPu, caudate putamen; NAc, nucleus accumbens. Scale bar is 2 mm; (**B**,**D**) Semi-quantification of Enk (**B**) and Dyn (**D**) mRNA levels (nCi/g) in the CPu after the above-mentioned treatments. Data represent mean ± SEM (*n* = 6). *p* > 0.05 (one-way ANOVA followed by Dunn’s test).

The effect of the six-day continuous treatment with morphine (20 mg/kg/day) and/or PD168,077 (1 mg/kg/day) on the expression of endogenous opioid peptides in the CPu was then studied by the determination of mRNA levels of Enk and Dyn using *in situ* hybridization. Autoradiograms showed in Figure 4 demonstrate that both Enk (Figure 4A–A3,B) and Dyn (Figure 4C–C3,D) mRNA levels were not affected by the different drug treatments.

## 3. Discussion

In the present work it was shown that six days of continuous administration of morphine produces an up-regulation of MOR IR and agonist binding in the striosomal compartment of the rat CPu. The results obtained in the semi-quantitative immunohistochemistry experiments indicate an increase of MOR protein level in the CPu. Furthermore, the saturation analysis of [^3^H]DAMGO binding using quantitative receptor autoradiography made it possible to demonstrate an increase in the density of MOR agonist binding sites in the striosomes, without changes in receptor affinity. The increase in MOR IR and agonist density was similar (by 40%) using these two experimental approaches, indicating that an increase in the density of functional MOR had taken place upon continuous morphine treatment. MOR is also expressed in the surrounding matrix [31], but the morphine-induced MOR up-regulation appeared to occur exclusively in the striosomes. It should be noted that MOR up-regulation was higher in the striosomes of the caudal CPu than in those in the rostral part. This regional specificity of the effect of continuous morphine treatment on MOR in striosomes correlates well with the rise of c-Fos in the caudal CPu [28], and provides new data to the concept of region-dependent regulation of striosomal neurons by opioids [32].

Continuous administration of morphine induces an increase in the basal values of [^35^S]GTPγS binding in the whole CPu *vs.* vehicle treated rats, reflecting an increase in G protein activity independent to the striatal compartments (matrix and striosomes). However, when MOR-mediated G protein activation was evaluated *in vitro* in rats which had previously been treated with continuous morphine, a higher degree of [^35^S]GTPγS binding was shown in the striosomes *vs.* vehicle after incubation with DAMGO, suggesting a specific regional manifestation of MOR sensitization. Thus, MOR activation by morphine could differentially modulate the striosomes *vs.* matrix compartment, leading potentially to an increased MOR modulation of their GABAergic output [33]. Together, these results suggest a positive relationship between the increase of MOR density and MOR-mediated G protein activation, known to involve mainly G_i/0_, likely leading to increased signal transduction efficiency.

MOR plays a key role in mediating morphine tolerance and dependence [22]. In fact, morphine pharmacological effects disappear in MOR knockout mice [5–7]. Tolerance to morphine has been proposed to result from down-regulation of MOR and/or decrease of MOR-mediated G protein activation or of effector activation. *In vitro* studies in cell culture models have clearly shown these MOR adaptations [22]. However, *in vivo* studies in the CNS have demonstrated a large variety of effect on MOR levels, including no changes [34], decrease [24,35], or increase [23,25,36]. Differences in the animal model employed, drug dosage, routes of administration, and the regions of the CNS analyzed could help explain the discrepancy in the results. Interestingly, our data are in good agreement with those from Viganò and colleagues [25], using different paradigms of morphine administration, *i.e.*, intermittent *vs.* continuous morphine treatment. By analyzing the behavioral responses of rats to the morphine treatment (catalepsy, non-stereotyped activity, and stereotyped activity), these authors related MOR up-regulation in the CPu to morphine sensitization [25], as also done in the current study. The dose of morphine (20 mg/kg/day, subcutaneous) used in the present study has been demonstrated to induce tolerance to its analgesic effect ([37] and own unpublished observation), which correlate with a decrease in MOR-mediated signaling, mainly in the spinal cord [38]. However, we demonstrate in the current work that the administration of morphine produces MOR sensitization, since an increase of MOR-mediated signaling has been shown in the striosomal compartment of the CPu. It should to be noticed that Fábián and colleagues [39] described an increase of the number of MOR-binding sites in the membrane microsomal fraction, which are enriched in endoplasmic reticulum, Golgi membranes, and endosomes, suggesting *de novo* synthesis of this opioid receptor. Therefore, the increase of MOR density which has been observed in the present work could be the result of MOR increases in both the plasma membrane and the microsomal fraction, and an increase in MOR mRNA levels induced by continuous morphine could be one mechanism involved. In contrast, Enk and Dyn mRNA levels are not altered by continuous morphine and seem to do not contribute to the MOR changes observed. These results are in agreement with our previous observation that changes in Enk and Dyn mRNA after the acute administration of morphine occur for only a short time and are transient [29]. However, it cannot be ruled out changes in opioid peptide levels are due to post-transcriptional regulation processes.

A large number of studies have described interactions between the dopaminergic and opioidergic systems. In fact, it was shown that dopamine receptors can modulate opioid receptor expression or its density in the cell surface membrane in multiple ways. While D_1_R increases MOR density in the cell surface membrane by the formation of a D_1_R-MOR heteromer [40], genetic deletion of the D_2_R down-regulates MOR density [41]. We have previously demonstrated that acute D_4_R stimulation down-regulates MOR IR in the striosomes of the CPu, suggesting that D_4_R interacts with MOR promoting its internalization and degradation [26]. The participation of other D2-like receptors, *i.e.*, D_2_R and D_3_R, has been ruled out since the acute treatment with quinpirole and raclopride were unable to induce MOR IR changes in the striosomal compartment [26]. Here, we have found that the continuous agonist stimulation of D_4_R produces the opposite effect, as a small but significant up-regulation of MOR IR has been observed in the striosomes of the caudal CPu, as well as a substantial increase of striosomal MOR density binding using the [^3^H]DAMGO agonist radioligand. The discrepancy between the degree of increase on MOR IR *vs.* [^3^H]DAMGO binding due to D_4_R agonist stimulation could be explained on the basis that the antibody against MOR which has been used in the present work recognizes both the active and inactive/intracellular conformation of the receptor, while [^3^H]DAMGO binding mainly reflects MOR-binding sites and thus functional MOR receptors in the plasma membrane. Thus, D_4_R may have a major role in the regulation of MOR trafficking, rather than *de novo* receptor synthesis. However, D_4_R activation *in vitro* did not affect DAMGO-stimulated [^35^S]GTPγS binding in the striosomes.

Nevertheless, the co-administration of continuous morphine and the D_4_R agonist PD168,077 fully counteracted the morphine-induced increases in striosomal MOR density and the enhancement of MOR-induced G protein activation. Thus, D_4_R activation may prevent development of the behavioral sensitization by morphine. Another important consequence of this antagonistic receptor-receptor interaction between D_4_R and MOR, which could take place in the plasma membrane (in receptor heteromer) and/or via changes in gene expression, could be the modulation of the GABA efferent projections from the striosomes. They innervate and modulate nigro-striatal dopamine neuron [20]. Thus, D_4_R could prevent massive release of dopamine from these dopamine neurons and thus the abnormal activation of striatal dopamine receptors. This view is supported by the D_4_R blocking effect on morphine-induced transcription factors in the CPu [28,29]. However, additional experiments are required to clarify this issue.

Antagonistic D_4_R and MOR receptor-receptor interactions in putative striosomal D_4_R-MOR heteromers have been proposed as one molecular mechanism for the D_4_R-MOR interaction observed. This was first indicated on the basis of anatomical data, which showed a co-localization of D_4_R and MOR in the striosomes [27], and later on by *in vitro* demonstration of a D_4_R modulation on MOR recognition and signaling [11,30]. This mechanism can best explain why the increase in MOR density by treatment with morphine and D_4_R agonist alone can be blocked by their combined treatment. Thus the two agonists by inducing bidirectional antagonistic allosteric receptor-receptor interactions in the putative heteromer can block the effects of each other. However, it should be stated that a D_4_R-MOR downstream crosstalk could also contribute to the results observed in the current study, which needs to be addressed in future works.

D_4_R and MOR functional interaction in the striosomes have a special interest because this striatal compartment has been related with habit learning and may also mediates the transition from impulsive to compulsive drug use [42–44]. Thus, impairment of D_4_R function especially in the striosomes could represent a factor for drug addiction vulnerability by producing dysfunction of the MOR protomer in the putative D_4_R-MOR heteromer of this striatal compartment.

## 4. Experimental Section

### 4.1. Animals

Adult male Sprague-Dawley rats (Charles River, Barcelona, Spain) weighing 250–300 g were used. Rats had continuous access to food and water and were maintained on a standard light/dark cycle (12/12 h) and constant room temperature (20 ± 2 °C) and relative humidity (65%–75%). Animal care and procedures described in the present study were in accordance with the guidelines of the Council of European Communities (86/609/EEC) as well as the Spanish Government (Real Decreto 1201/2005) and all efforts were made to minimize animal suffering and to reduce the number of animals used.

### 4.2. Drugs

Morphine sulphate was obtained from the Ministerio de Sanidad, Servicios Sociales e Igualdad (Spanish Government). PD168,077 maleate (D_4_R agonist) and L745,870 trihydrochloride (D_4_R antagonist) were obtained commercially (Tocris Bioscience, Avonmouth, UK). PD168,077 has been proven not to interact with MOR (see Supplementary Information). All drugs were dissolved in a vehicle solution consistent of 2% dimethyl sulfoxide (DMSO) in 0.9% NaCl. We have previously demonstrated that this amount of DMSO exerts no effect on receptor function [26].

### 4.3. Drug Administration

Rats received continuous administration of vehicle, morphine (20 mg/kg/day), PD168,077 (1 mg/kg/day) and L745,870 (1 mg/kg/day), alone or in combination by an osmotic pump (2ML1, rate of release: 10 μL/h, 7 days delivery, Alzet^®^ osmotic pumps, Cupertino, CA, USA) that was subcutaneously implanted under deep anaesthesia (75 mg/kg ketamine and 0.5 mg/kg medetomidine) between the shoulder blades. Treatment duration was 6 days.

### 4.4. Immunohistochemistry

Rats (*n* = 5 per treatment) were anesthetized with sodium pentobarbital (60 mg/kg, intraperitoneal) and perfused transcardially with 0.1 M phosphate-buffered saline, pH 7.4 (PBS), followed by 4% paraformaldehyde (*w*/*v*) in 0.1 M phosphate buffer, pH 7.4 (PB). The brains were rapidly removed and postfixed in the same fixative overnight at 4 °C, cryoprotected in 30% sucrose in PBS for 72 h and frozen in dry ice. Free-floating coronal sections (30 μm thick) were obtained with a freezing microtome (CM 1325; Leica, Wetzlar, Germany) and endogenous peroxidase activity was quenched by incubation for 15 min with 3% H_2_O_2_ in PBS. Sections were first incubated with a rabbit polyclonal anti-MOR antibody (Calbiochem, Germany) diluted 1:1000 in PBS with 0.2% Triton X-100 (PBS-TX) and 0.1% sodium azide for 48 h at RT, then in biotin-conjugated goat anti-rabbit IgG diluted 1:500 in PBS-TX (Vector Laboratories, Burlingame, CA, USA) for 1 h and finally in streptavidin-peroxidase complex (Sigma-Aldrich, St. Louis, MO, USA) diluted 1:2000 in PBS-TX for 1 h. Peroxidase activity was visualized with 0.05% 3,3′-diaminobenzidine (DAB, Sigma-Aldrich, St. Louis, MO, USA) and 0.002% H_2_O_2_, and staining was intensified with 0.8% nickel ammonium sulfate. The sections were then mounted on gelatin-coated slides, air dried, dehydrated with ethanol, cleared in xylene and coverslipped with DPX-mounting medium.

Semi-quantitative analysis of MOR IR intensity was performed as we have described and validated previously using the NIH Image J system [26]. Briefly, MOR IR O.D. was measured from microphotographs obtained with a digital camera (Coolpix 4500, Nikon, Tokyo, Japan) under light microscopy (Nikon E400, Nikon, Tokyo, Japan). The O.D. values from the striosomes and matrix compartments were obtained bilaterally from rostral, middle, or caudal levels of the CPu and they were corrected with the O.D. from an immunonegative area.

### 4.5. μ Opioid Receptor Autoradiography

Rats (*n* = 6 per treatment) were sacrificed by decapitation and the brains were rapidly removed, frozen by immersion in dry ice-cooled isopentane (−30 °C) and stored at −80 °C until sectioning. Coronal brain sections (14 μm) at the CPu level were cut on a cryostat (Microm HM 550, Microm Laborgerate S.L., Barcelona, Spain), thawed onto glass slides and stored at −20 °C until use. Autoradiographic saturation kinetic study of MOR was performed using [^3^H]DAMGO as radioligand (specific activity 56 Ci/mmol; PerkinElmer, Waltham, MA, USA). The sections were pre-incubated for 30 min at RT with 50 mM Tris-HCl (pH 7.4) and 5% BSA to remove endogenous opioids, and then incubated for 1 h with the same buffer containing [^3^H]DAMGO (concentration ranging from 0.36 nM to 4 nM). Adjacent sections were used for control, in which non-specific binding was defined as the [^3^H]DAMGO binding in the presence of 10 μM of naloxone (Tocris Bioscience, Avonmouth, UK). After incubation with [^3^H]DAMGO, sections were sequentially washed for 5 min, each in ice-cold Tris-HCl buffer (two times) and distilled water (one time), and air-dried. Thereafter, the sections were exposed to a tritium-sensitive film (BioMax MR Film, Kodak, Rochester, NY, USA) for 6 weeks, together with prefabricated ^3^H-labeled polymer standard strips (GE Healthcare, Piscataway, NJ, USA). The films were revealed and digitalized (ScanMaker 9800XL, Microtek International Inc., Santa Fe Springs, CA, USA). Quantitative measurements of autoradiographic signals (grey values) were made using the analyzing system ImageJ 1.44p (NIH, Bethesda, MD, USA). Measurements were made bilaterally from the striosomes and matrix compartment of the CPu. Grey values were corrected for the contribution due to non-specific binding and converted into fmol/mg proteins using the ^3^H-standards described above. Data were analyzed by nonlinear regression analysis (GraphPad Prism 5, GraphPad Software, Inc., La Jolla, CA, USA) for the determination of *B*_max_ and *K*_d_ values.

### 4.6. Agonist-Stimulated [^35^S]GTPγS Binding in Autoradiography

Sections from rat brains (*n* = 4–5 per treatment) were obtained as described before for the MOR autoradiography experiment. The sections were pre-incubated in assay buffer (50 mM Tris-HCl, 3 mM MgCl_2_, 2 μM EGTA, 100 μM NaCl, pH 7.4) for 15 min at RT and then in 2 mM GDP in assay buffer for 15 min. Sections were then transferred into assay buffer containing 0.2 mM GDP and 0.04 nM [^35^S]GTPγS with (stimulated) or without (basal) 3 μM DAMGO and/or 90 nM PD168,077 and incubated for 40 min at RT. Finally, the sections were rinsed twice in cold 50 mM Tris-HCl buffer and once in distilled water, dried and exposed to BioMax MR Film (Kodak, Rochester, NY, USA) for 48 h. Films were developed and digitized as described above. Gray values were measured from each section using the analyzing system ImageJ 1.44p (NIH, Bethesda, MD, USA), corrected by subtraction of the background gray value and converted to nCi/g using a ^14^C standard (GE Healthcare, Piscataway, NJ, USA).

### 4.7. *In Situ* Hybridization

For the detection of Enk and Dyn mRNA using *in situ* hybridization, rats (*n* = 6 per treatment) were sacrificed by decapitation. The brains were removed, frozen in dry ice-cooled isopentane, and stored at −80 °C. Coronal sections (14 μm thick) at the CPu level were obtained on a cryostat (Microm HM 550, Microm Laborgerate S.L., Barcelona, Spain), thawed onto glass slides and stored at −20 °C until incubation. Detection of prodynorphin mRNA (296–345) [45] and preproenkephalin mRNA (235–282) [46] was made using 48-mer oligonucleotides complementary to described nucleotides. The probes were 3′-end labelled with [α-^33^P]dATP (PerkinElmer, Waltham, MA, USA) using terminal deoxynucleotidyl transferase (GE Healthcare, Piscataway, NJ, USA). The hybridization cocktail contained 50% formamide, 4× SSC (1× SSC is 0.15M NaCl, 0.0015M sodium citrate; pH 7.0), 1× Denhardt’s solution, 1% sarcosyl, 0.02M Na_3_PO_4_ pH 7.0, 10% dextransulphate, 0.06 M DTT and 0.1 mg/mL of sheared salmon sperm DNA. Hybridization reaction was performed for 16–18 h in a humidified chamber at 42 °C. After hybridization, sections were rinsed four times (20 min each) in 1× SSC at 60 °C and one time at RT. Finally, sections were rinsed in autoclaved water, dehydrated in alcohol and air dried. Thereafter, the sections were exposed to film (Kodak BioMax MR-1, GE Healthcare, Piscataway, NJ, USA) for 3 days. Autoradiogram films were digitized and O.D. values from the CPu were determined using the NIH ImageJ system. A ^14^C standard (GE Healthcare, Piscataway, NJ, USA) was used to correlate O.D. readings on the autoradiograms to amount of radioactivity (nCi/g).

### 4.8. Statistical Analysis

Statistical analysis was made with one-way or two-way analysis of variance (ANOVAs), followed by Dunn or Bonferroni *post hoc* test. Statistical significance was set at *p* < 0.05, *p* < 0.01 and *p* < 0.001.

## 5. Conclusions

D_4_R activation during continuous treatment with morphine counteracts the induced changes on MOR protein levels and receptor signaling in the striosomes of the rat CPu.

## Supplementary Information

### 1. Methods

#### 1.1. Cell Culture and Transfection

HEK293T cells were cultured in DMEM (Dulbecco’s modified Eagle’s medium) supplemented with 10% (*v*/*v*) fetal calf serum with l-glutamine (2 mM), penicillin (100 U mL^−1^) and streptomycin (0.1 mg mL^−1^), in a controlled environment (37°C, 98% humidity, 5% CO_2_). For transfection, cells were grown until 50% confluency. Next, cells were transiently transfected with plasmids encoding HA-D4.2R or FLAG-MOR using the calcium phosphate transfection method (using 10 μg DNA per 10 cm dish or 2 μg per 6-well)) as described previously [47].

#### 1.2. ERK1/2 Phosphorylation Assay on Western Blot

48-h post-transfection cells were starved overnight with DMEM medium without fetal calf serum. Cells were then incubated for 5 min with DAMGO (Sigma-Aldrich, St. Louis, MO, USA) or PD168,077 (Tocris, Bristol, UK) in concentrations ranging from 10^−5^ to 10^−10^ M in DMEM medium at 37 °C. Incubation was stopped by washing the cells two times with ice-cold PBS and putting them on ice. Subsequently cells were lysed with 200 μL SDS-sample buffer (62.5 mM Tris/HCl pH 6.8; 2% SDS; 10% glycerol; 0.1% bromophenol blue; 50 mM dithiothreitol) per 6-well. Samples were sonicated for 1 min and heated for 5 min at 95 °C. The lysates were loaded on a 10% SDS-PAGE gel. Proteins were transferred onto a nitrocellulose membrane. Next, membranes were blocked with blocking buffer (1:1 Licor blocking buffer/TBS) for 1 h. Membranes were then incubated for 1 h with primary antibodies rabbit phospho-p42/p44 (Cell signaling, Danvers, MA, USA) and mouse p42/p44 (Cell signaling, Danvers, MA, USA) in 1:1 Licor blocking buffer-TBST. Afterwards, the blots were incubated with secondary antibodies 1:20,000 anti-mouse redLT (LI-COR Biosciences, Lincoln, NE, USA) and 1:15,000 anti-rabbit green (LI-COR Biosciences, Lincoln, NE, USA) for 1 h and developed with the Odyssey Imaging System (OdysseyV3.0, Westburg, Leusden, The Netherlands).

#### 1.3. In-Cell Western ERK1/2 Phosphorylation Assay

The day after transfection, cells were reseeded on poly-d-lysine (Sigma-Aldrich, St. Louis, MO, USA) coated 96-well plates. These cells were grown until 90% confluency after which they were starved for 2 h with DMEM medium without fetal calf serum. Cells were then incubated for 5 min with DAMGO (Sigma-Aldrich, St. Louis, MO, USA) or PD168,077 (Tocris, Bristol, UK) in concentration ranging from 10^−5^ to 10^−10^ M in DMEM medium at 37 °C. Incubation was stopped by removing the culture medium, followed by addition of fixing solution (3.7% formaldehyde in PBS) for 20 min at RT. Subsequently cells were permeabilized, by washing 5 times for 5 min with Triton washing solution (0.1% Triton X-100 in PBS). Cells were blocked with LI-COR Odyssey blocking buffer (LI-COR Biosciences, Lincoln, NE, USA) for 90 min with moderate shaking on a rotator, after which the cells were incubated overnight at 4 °C with two primary antibodies: 1:1000 rabbit phospho-p42/p44 (Cell signaling, Danvers, MA, USA) and 1:800 mouse p42/p44 (Cell signaling, Danvers, MA, USA) diluted in blocking buffer. The plate is washed at RT for 5 times 5 min with Tween washing solution (0.1% Tween-20 in PBS). Fluorescently labeled secondary antibodies: 1:800 anti-mouse redRD (LI-COR Biosciences) and 1:800 anti-rabbit green (LI-COR Biosciences, Lincoln, NE, USA) are added to the cells and incubated at RT for 1 h. After final washing steps, the plate is measured with the Odyssey Infrared Imaging system (LI-COR Biosciences, Lincoln, NE, USA). For analysis, background values for the secondary antibody are subtracted from the values and the phospho-p42/p44 signal is normalized against the total p42/p44 signal.

### 2. Results

#### 2.1. Specificity of the Drug PD168.077 for the Dopamine D_4_ Receptor

Dopamine D_4_ receptors and MOR are known to activate the mitogen-activated protein kinase (MAPK) signalling pathway, resulting in phosphorylation of p42/p44 [48,49]. Therefore, to investigate whether the dopamine D_4_ receptor agonist PD168,077 can act as an agonist or an inverse agonist of MOR, we performed two kinds of MAPK-phosphorylation assays, *i.e.*, a Western blot immunodetection assay and an in-cell Western assay. First, HEK293T cells were transiently transfected with pHAD_4.2_R (as a positive control) and pFLAGMOR. Next, receptors were stimulated with different concentration of DAMGO (as a positive control for MOR expression) and of PD168,077 and phosphorylation of p42/p44 was determined (Figure S1). From both experiments, the Western blot immunodetection and the in-cell Western assay, we can conclude that PD168.077 activates the MAPK pathway when the dopamine D_4_ receptor is expressed and this already occurs at concentrations as low as 0.1 nM (Figure S1B). There is a small activation of p42/p44 upon MOR stimulation but only at high concentration (1 to 10 mM). The MOR is functionally expressed as DAMGO stimulates p42/p44 phosphorylation. Next, also DAMGO activates the dopamine D_4_ receptor at the highest concentrations (1 to 10 mM). The concentrations used to indicate that PD168,077 counteracts morphine-induced changes in MOR signaling are much lower (90 nM). Therefore we believe that the effect of PD168,077 on signaling via MOR is very low or even negligible.

Figure S1. Specificity PD168,077 for dopamine D_4_ receptor. (**A**) Western blot analysis of MAPK phosphorylation. HEK293T cells transiently expressing HAD_4.2_R or FLAGMOR were treated for 5 min with different concentrations of DAMGO or PD168.077 (PD) (10^−9^ to 10^−5^ M). Cell lysates were made and a Western immunoblot with was performed using the primary antibodies rabbit phospho-p42/p44 and mouse p42/p44; (**B**) In-cell western P-MAPK assay. An in-cell western assay was performed on HEK293T cells transiently expressing HAD_4.2_R or FLAGMOR. Cells were treated for 5 min with DAMGO or PD168.077 in a concentration range from 10^−10^ to 10^−5^ M. Phosphorylated p42/p44 values were normalized to total p42/44. Red = D_4.2_R + DAMGO; black = D_4.2_R + PD168077; blue = MOR + DAMGO; green = MOR + PD168077; DAMGO (agonist of MOR); PD168.077 (specific agonist of D_4_R).

## Figures and Tables

**Table 1.** Effect of six days of continuous treatment with morphine (20 mg/kg/day), PD168,077 (1 mg/kg/day) and L745,870 (1 mg/kg/day) alone or combined on mu opioid receptor (MOR) immunoreactivity (IR) in the striosomes throughout the rostro-caudal axis of the rat CPu.

Level of the CPu	Vehicle	Morphine	PD168,077	L745,870	Morphine + PD168,077	Morphine + PD168,077 + L745,870
Rostral	100 ± 2.3	**134.6 ± 3.2**	109.6 ± 3.1	91.4 ± 3.4	99.4 ± 3.4	**135.0 ± 4.1**
Middle	100 ± 2.6	**133.8 ± 3.6**	104.2 ± 3.5	100.4 ± 4.0	98.0 ± 4.2	**127.4 ± 5.0**
Caudal	100 ± 2.9	**169.4 ± 6.1** ^*^	**117.6 ± 4.6** ^*^	111.5 ± 6.9	98.6 ± 5.7	149.0 ± 6.2^*^

Mean optical density values (mean ± SEM; *n* = 5) are given as percentage of control. Bold numbers indicate a statistically significant difference with respect to the corresponding vehicle-treated group in each rostro-caudal level of the CPu (one-way ANOVA followed by *post hoc* Dunn’s multiple comparison test; *p* < 0.05). Asterisks indicate a statistically significant difference between the rostral, middle and caudal levels of the CPu in each drug-treated group (one-way ANOVA followed by *post hoc* Bonferroni *t*-test; *p* < 0.05).
