# Supplementary figures and images for: Dopamine D4 Receptor Counteracts Morphine-Induced Changes in μ Opioid Receptor Signaling in the Striosomes of the Rat Caudate Putamen
Source: Int J Mol Sci. 2014 Jan 21;15(1):1481–98. doi: 10.3390/ijms15011481 (PMC3907881; doi:10.3390/ijms15011481)

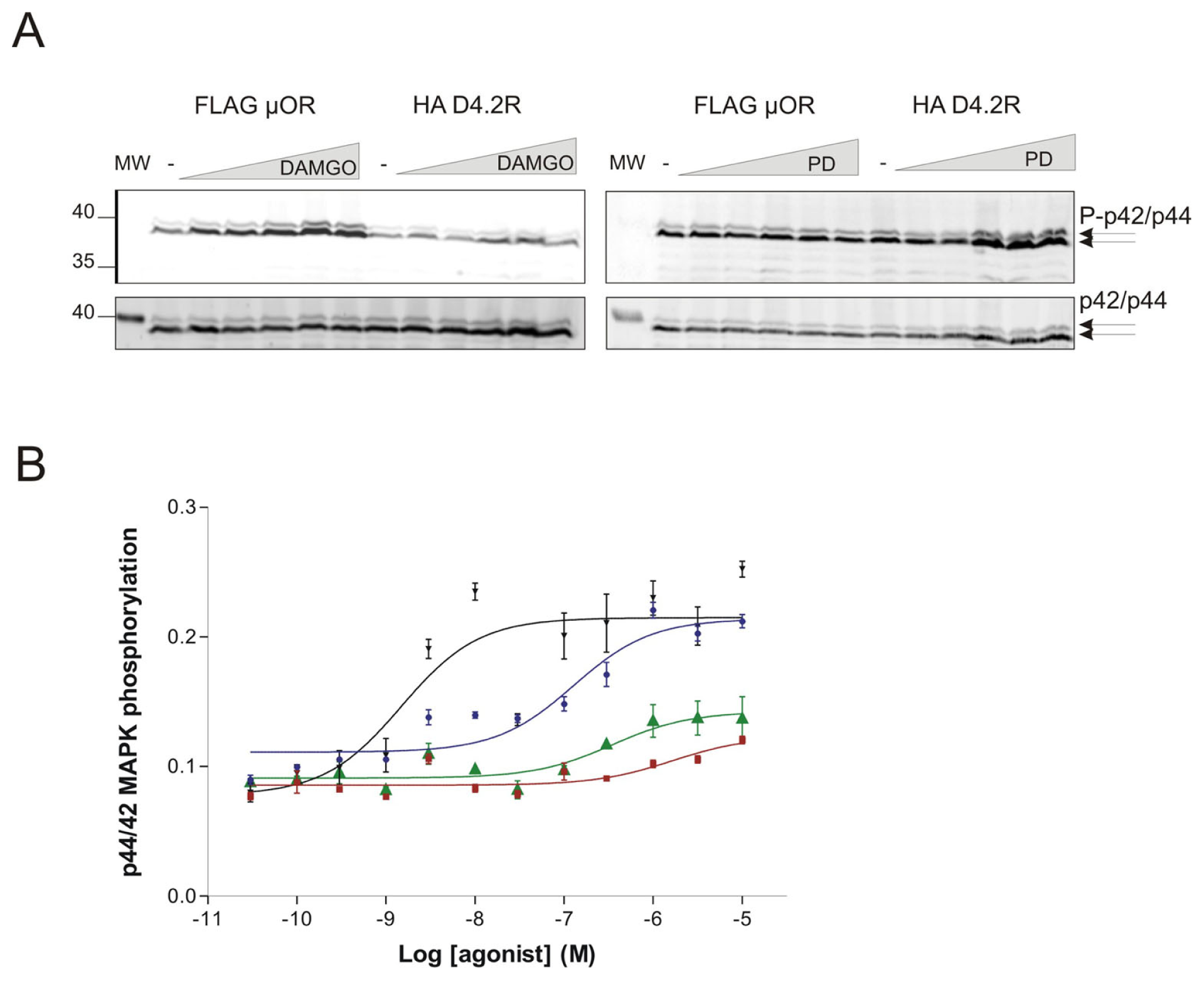

Supplement: Figure S1. — Specificity PD168,077 for dopamine D4 receptor. (A) Western blot analysis of MAPK phosphorylation. HEK293T cells transiently expressing HAD4.2R or FLAGMOR were treated for 5 min with different concentrations of DAMGO or PD168.077 (PD) (10−9 to 10−5 M). Cell lysates were made and a Western immunoblot with was performed using the primary antibodies rabbit phospho-p42/p44 and mouse p42/p44; (B) In-cell western P-MAPK assay. An in-cell western assay was performed on HEK293T cells transiently expressing HAD4.2R or FLAGMOR. Cells were treated for 5 min with DAMGO or PD168.077 in a concentration range from 10−10 to 10−5 M. Phosphorylated p42/p44 values were normalized to total p42/44. Red = D4.2R + DAMGO; black = D4.2R + PD168077; blue = MOR + DAMGO; green = MOR + PD168077; DAMGO (agonist of MOR); PD168.077 (specific agonist of D4R). [file ijms-15-01481s1.tif]
